# The potential of converting carbon dioxide to food compounds *via* asymmetric catalysis

**DOI:** 10.1039/d3na00178d

**Published:** 2023-05-05

**Authors:** Rui Gao, Xinxin Xu, Zhimeng Wu, Liguang Xu, Hua Kuang, Chuanlai Xu

**Affiliations:** a International Joint Research Laboratory for Biointerface and Biodetection, Jiangnan University Wuxi Jiangsu 214122 PRC; b State Key Laboratory of Food Science and Technology, Jiangnan University Jiangsu PRC; c The Key Laboratory of Carbohydrate Chemistry & Biotechnology, Ministry of Education, School of Biotechnology, Jiangnan University Wuxi Jiangsu 214122 PRC; d The Key Laboratory of Synthetic and Biological Colloids, Ministry of Education, School of Chemical and Material Engineering, Jiangnan University Wuxi Jiangsu 214122 PRC xxx89826@163.com

## Abstract

The food crisis caused by diminished arable land, extreme weather and climate change linked to increased carbon dioxide (CO_2_) emission, is threatening global population growth. Interestingly, CO_2_, the most widespread carbon source, can be converted into food ingredients. Here, we briefly discuss the progress and challenges in catalytic conversion of CO_2_ to food ingredients *via* chiral catalysis.

## Abstract 2

An artificial starch anabolic pathway (ASAP) system that converts CO_2_ to starch.

## Graphical abstract

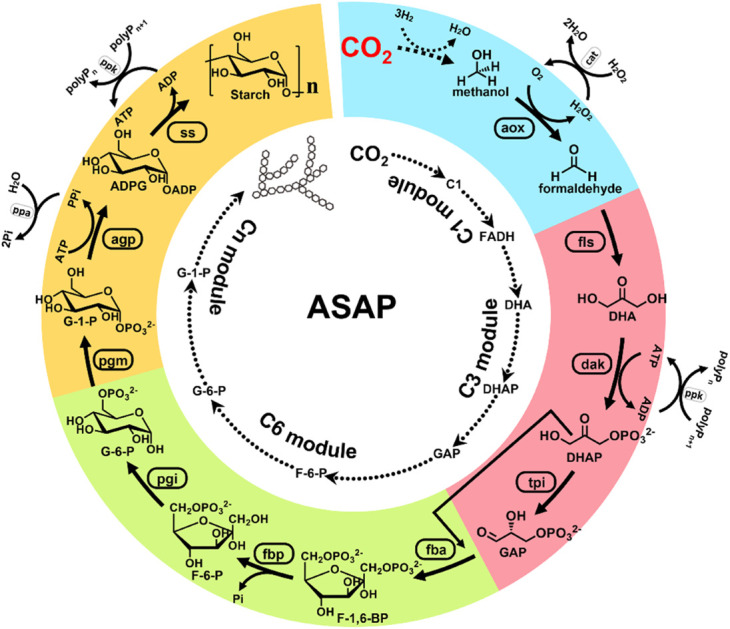

## Biocatalytic conversion of CO_2_ to food

The projection that the global population will increase to 9.7 billion by 2050 poses a higher demand for food production. Specifically, agricultural production needs to increase by up to 60% in order to feed the increased population.^1^ However, the diminished arable land, and the increased CO_2_ emission amounts which lead to extreme weather and climate change make this goal more intangible.^1^ CO_2_, a well-known greenhouse gas, can serve as the most widespread and abundant carbon resource. Using CO_2_ as a raw material for synthesizing typical food and its ingredients such as chiral amino acids and carbohydrates can not only reduce CO_2_ emissions, but also alleviate the food crisis caused by rapid population growth. In this regard, nature uses photosynthesis to fix carbon dioxide and turn it into food.

**Fig. 1:**
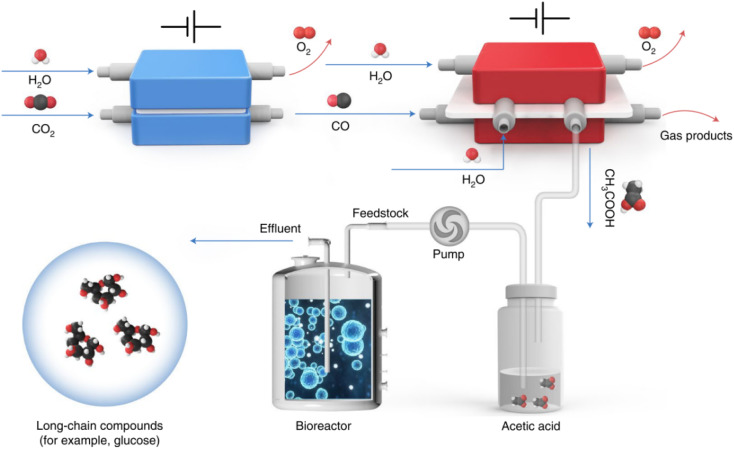
Schematic illustration of an artificial synthesis system for glucose. CO_2_ was first converted to acetic acid through a two-step electrochemical reaction. The generated acetic acid was then fermented in a bioreactor as a microbial substrate to produce long-chain compounds such as glucose (ref. 2).

Recently, Zheng *et al.* developed a hybrid electro-biosystem by coupling spatially separated CO_2_ electrolysis with yeast fermentation, which efficiently converts CO_2_ to d-glucose or fatty acids.^2^d-Glucose is the predominant form of carbohydrates utilized by human beings, so the synthesis of d-glucose is very important in the field of food manufacture. In this interesting study, the researchers employed three steps to achieve the synthesis of long-chain energy substances from CO_2_. First, Ni-based single-atom catalysts were used to realize almost 100% CO_2_ electroreduction to CO which was efficiently converted to acetic acid in the second step by using a grain-boundary-rich Cu catalyst. In this step, a porous solid electrolyte reactor was developed in order to obtain high-purity acetic acid and avoid complex isolation and purification steps. Finally, high-purity acetic acid was utilized as the feedstock for genetically engineered *Saccharomyces cerevisiae* fermentation, which delivers an average d-glucose yield of 8.90 μmol per gram of yeast per hour. In addition to d-glucose, the production of other valuable products was also showcased by taking advantage of this platform. For example, using a similar design, the researchers engineered strain LXJ015 to electrically produce free fatty acids *in vitro* from pure acetic acid (Fig. 1).^2^

**Fig. 2:**
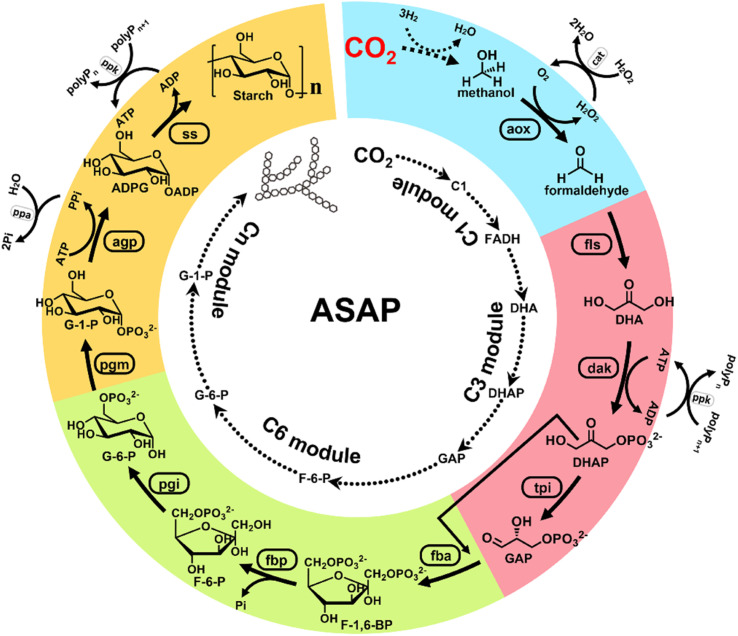
Design of an artificial starch anabolic pathway (ASAP) system that converts CO_2_ to starch. CO_2_ is first reduced to one-carbon units (C1), such as methanol. Then, methanol is oxidized to formaldehyde (FADH). FADH is treated with formolase (FLS) to produce dihydroxyacetone (DHA). Then, DHA is phosphorylated to form dihydroxyacetone phosphate (DHAP). DHAP is treated with triose phosphate isomerase (TPI) to from d-glyceraldehyde 3-phosphate (GAP). So far, the synthesis of three-carbon units (C3) has been completed. Next, GAP is treated with (FBA) to form d-fructose-1,6-bisphosphate (F-1,6-BP). Then, F-1,6-BP is treated with fructose-bisphosphatase (FBP) to form d-fructose-6-phosphate (F-6-P). F-6-P is then treated with phosphoglucose isomerase (PGI) to form d-glucose-6-phosphate (G-6-P). The presence of G-6-P implies the successful preparation of six-carbon units (C6). After that, G-6-P is treated with phosphoglucomutase (PGM) to form d-glucose-1-phosphate (G-1-P). Then, G-1-P is treated with ADP-glucose pyrophosphorylase to form ADP glucose (ADPG). ADPG then is treated with starch synthase (SS) to form starch. This is the final composite module (C*n*) of the ASAP system (ref. 3).

Earlier, Cai *et al.* reported a chemical–biochemical hybrid pathway for the synthesis of starch from CO_2_ and hydrogen in a cell-free system through 11 core enzyme-catalyzed reactions.^3^ Starch is the most important carbohydrate utilized by people. In this research, CO_2_ was first reduced to a C1 compound (formic acid or methanol) using a chemical catalyst. The C1 compound was then utilized as a substrate for the artificial starch anabolic pathway (ASAP) system developed by the same researchers. In the ASAP system, they divided the starch synthesis into a C1 module (for formaldehyde production), C3 module (for d-glyceraldehyde-3-phosphate production), C6 module (for d-glucose-6-phospate production), and C*n* module (for starch synthesis). The enzymatic processes were delicately combined with the chemical catalysis processes, and each module of the enzymatic processes was optimized by mutating the core enzymes of catalytic pathways, namely, formolase, fructose-bisphosphatase, and ADP-glucose pyrophosphorylase. Finally, an ASAP 3.0 system, composed of C1e, C3a, C6b*, and C*n*b modules, was determined with 10 enzyme-catalyzed reactions using methanol as the substrate. This system delivered a remarkably high starch productivity of ∼410 mg L^−1^ h^−1^ from CO_2_. This landmark work provides for the first time a way to convert waste CO_2_ into an economically valuable food ingredient *via* a combination of chemical catalysis, biosynthesis, and protein engineering (Fig. 2).^3^

As an emerging area in CO_2_ utilization, the two studies mentioned above not only open new strategies for preparing food products by combining electrochemistry with biocatalysis, but also provide potential solutions to alleviate the food crisis. Nonetheless, both studies synthesize chiral food ingredients from CO_2_ by taking advantage of chiral biological systems. Strictly speaking, it is not a direct route to transform CO_2_ to chiral food ingredients, since two separate processes, chemical transformation and biological transformation, were integrated in both studies. The former harvests achiral C1 compounds or C2 compounds, while the latter delivers chiral compounds by utilizing the asymmetric catalytic transformation function of biological systems.

So far, the chemo-catalytic conversion of CO_2_ into chiral compounds with specific configurations, especially chiral food components (amino acids, monosaccharides, and fatty acids), has been slow to develop. This could be attributed to the following challenges that hinder the development of an operable technology for the direct asymmetric conversion of CO_2_. First, CO_2_ is a very stable and inert molecule since it is in the lowest energy state, and the carbon in CO_2_ is in its most stable valence (IV). Second, it is rather difficult for CO_2_ to approach metal catalysts because of its weak coordination ability with metal catalysts and low solubility in non-alkaline solvents. Third, direct asymmetric conversion of achiral CO_2_ into chiral food ingredients requires a complex and tedious organic chemical synthesis process, and the derived products from the catalytic processes are often mixed enantiomers, which require an expensive enantiomeric sieving process. Furthermore, because of the inert nature of CO_2_, its catalytic conversion usually requires high temperature and high pressure, which are not conducive to the preparation of chiral compounds with specific configurations.^4^

In conclusion, chemical methods for the catalytic conversion of CO_2_ into valuable chiral chemicals are feasible, but the synthesis of chiral food components still requires process optimization and cost reduction, which inspire us to search for efficient catalytic pathways and chiral catalysts for this challenging chemical transformation.

## Asymmetric catalysis

Asymmetric catalysis, also known as chiral catalysis, renders catalytic reactions to proceed in the direction of the mirror isomers that match chiral catalysts, thereby enabling the controlled synthesis of chiral compounds with various specific configurations. Because of the chiral amplification effect, it is one of the most effective methods for synthesizing chiral products.^5^ The key to chiral catalytic reactions is the development of efficient chiral catalysts. In recent years, due to the combined amplification effect^6^ of chirality in asymmetric catalysis with the excellent opto-electromagnetic properties of nanomaterials,^7^ chiral nanomaterials have received increasing attention,^8^ and great progress has been made in the field of asymmetric catalysis.^9^ For example, Zhang *et al.* reported versatile and efficient asymmetric cross-coupling between carbonyl radicals (from aldehydes) and aryl radicals (from aryl halides) by utilizing porous chiral metal–organic frameworks as catalysts under light illumination.^10^ Wei *et al.* accomplished the dimerization of a specific configuration of the prochiral molecule 2-anthracenecarboxylic acid under light by controlling the chirality of metal nanohelices which were produced by glancing-angle deposition on the substrates rotated in the clockwise or counterclockwise direction.^11^ Chiral nanomaterials also exhibit fascinating asymmetric catalytic functions relevant to biocatalysis.^12^ In addition, compared with enzymes, nanomaterials cost less and have good environmental stability. Importantly, nanomaterial catalytic products often require only simple centrifugal separation to obtain high purity of the targets. Our group has demonstrated that chiral CdTe quantum dots with the function of restriction endonuclease can specifically cut DNA, and the cleavage efficiency is dependent on the configuration of the chiral CdTe quantum dots.^13^ Chiral Cu_1.96_S nanoparticles (NPs) and Cu_2_S NPs have been reported to display chiral-configuration-dependent cleavage capability for the tobacco mosaic virus capsid protein^9^ and hepatitis B core antigen,^14^ respectively. Chiral nanoparticles can also catalyze the polymerization of some biomolecules. Li *et al.* reported that chiral nanoscale inorganic superparticles can serve as photocatalysts that enantioselectively catalyze l-tyrosine and d-tyrosine into l-dityrosine and d-dityrosine, respectively.^12^

Therefore, asymmetric catalysis based on chiral nanomaterials is thriving and could become an important direction for the direct asymmetric catalytic conversion of CO_2_ to chiral food ingredients. Nonetheless, catalytic conversion of CO_2_ into chiral food components inevitably involves complex processes including carbon chain elongation, polymerization, amination, sulfurization, *etc.* Access to readily available and easily convertible chiral intermediates is the key to the direct synthesis of food ingredients from CO_2_. Next, we briefly describe how to obtain potential chiral intermediates from CO_2_ since they are indispensable for the preparation of more complex chiral food components.

## Progress in direct catalytic conversion of CO_2_ into chiral intermediates

To date, asymmetric catalytic conversion of CO_2_ into chiral intermediates can be mainly divided into the categories of asymmetric C–O bonds, asymmetric C–N bonds, asymmetric C–C bonds, and asymmetric C–H bonds on the basis of the products formed.

**Fig. 3:**
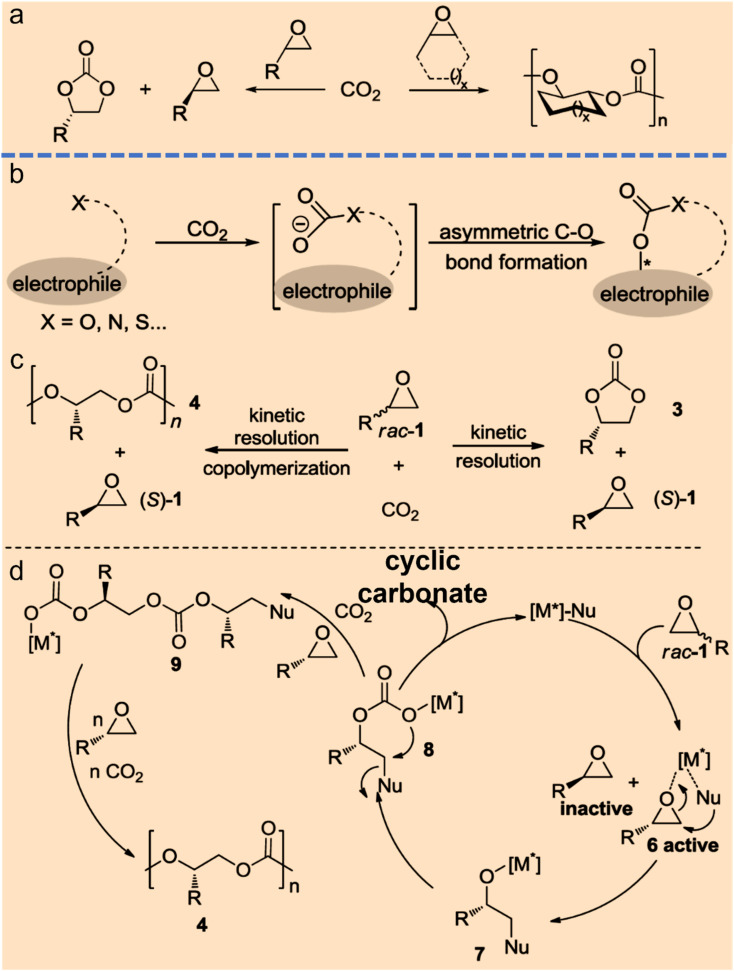
Strategies for direct catalytic conversion of CO_2_ into chiral intermediates. Asymmetric C–O bond formation with CO_2_*via* kinetic resolution (a) or nucleophiles (b) (ref. 4). (c and d) Chiral metal complex and nucleophile co-catalyzed kinetic resolution of racemic epoxides with CO_2_ to form chiral cyclic carbonates and polycarbonates (ref. 4).

The formation of asymmetric C–O bonds refers to the bonding method where the carbon atoms of CO_2_ become chiral carbon atoms after the addition of oxygen from reactants. The formation of asymmetric C–O bonds can be achieved by either asymmetric nucleophilic attack on CO_2_ by O-nucleophiles^15^ or kinetic resolution of epoxides with CO_2_.^16^ Strategies for constructing chiral C–O bonds directly on carbon radical centers are attractive, but the control over stereoselectivity remains a formidable challenge. Zhu *et al.* reported a copper-catalyzed regioselective and enantioselective carboesterification of substituted dienes using alkyl diacyl peroxides as the source of both carbon and oxygen substituents. This work represents a striking advance in the development of asymmetric radical C–O bonds and may lead to the discovery of other asymmetric radical reactions.^15^ Lu *et al.* found that chiral cyclic carbonates can be synthesized by employing cobalt(iii)-based complexes as chiral catalysts and the nucleophiles as cocatalysts during the kinetic resolution of racemic propylene oxide and CO_2_.^17^ Jing *et al.* realized the kinetic resolution of epoxides and CO_2_ with an *ee* value of 52% by loading Ni-based chiral catalyst into a three-dimensional porous metal–organic framework and using *n*-Bu_4_NBr as a cocatalyst.^18^ North *et al.* declared that cyclic carbonates can be synthesized over a dinuclear chiral catalyst consisting of a chiral Al(iii)-based catalyst and a Cr(iii)-based catalyst in the presence of *n*-Bu_4_NBr as a cocatalyst.^19^ Johnston and coworkers developed an elegant strategy to produce chiral cyclic carbonates *via* the carboxylation/alkene functionalization reaction of homoallylic alcohols. They also confirmed that a properly balanced Brønsted acid/base bifunctional catalyst lowers the barrier of CO_2_ incorporation and facilitates the stabilization of the resulting adduct, which are essential for the nucleophilic addition of a nucleophile to CO_2_ (Fig. 3).^20^

**Fig. 4:**
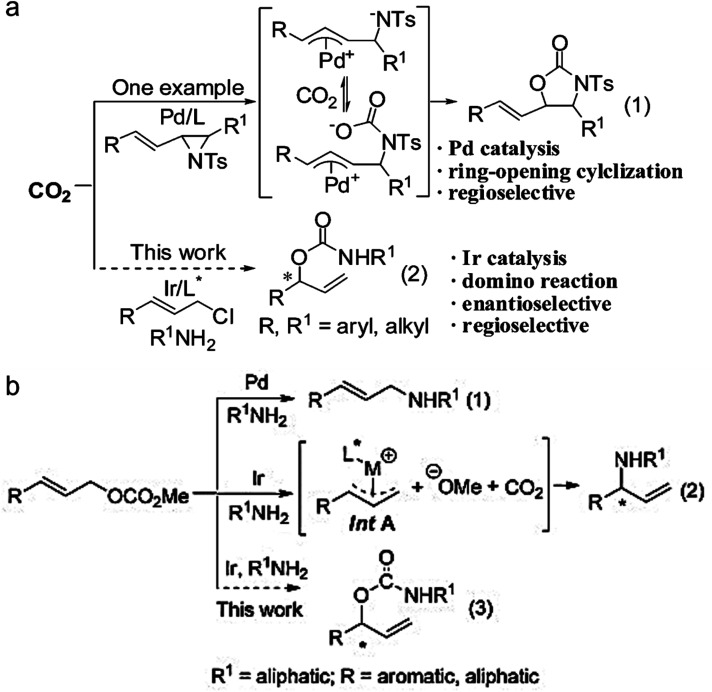
(a) Domino reactions involving Pd or Ir-catalyzed allylation reaction (ref. 23). (b) Transition-metal-catalyzed allylation of allyl carbonates with amines (ref. 24).

The formation of asymmetric C–N bonds implies that the carbon atoms of CO_2_ become chiral following the nitrogen addition reaction. The asymmetric C–N bond formation can be realized by either asymmetric nucleophilic attack on CO_2_ by N-nucleophiles^21^ or asymmetric carboxylation of amines and their derivatives with CO_2_ using chiral catalysts.^22^ In the former case, Zhang *et al.* synthesized chiral allyl carbamate from CO_2_ by the catalytic domino reaction for the first time with a high *ee* value of 94%.^23^ In the latter case, Zheng *et al.* found that the nucleophilic addition of CO_2_ using amine nucleophiles can enantioselectively convert allyl carbonate to branched-chain carbamate with an *ee* value up to 93% (Fig. 4).^24^

**Fig. 5:**
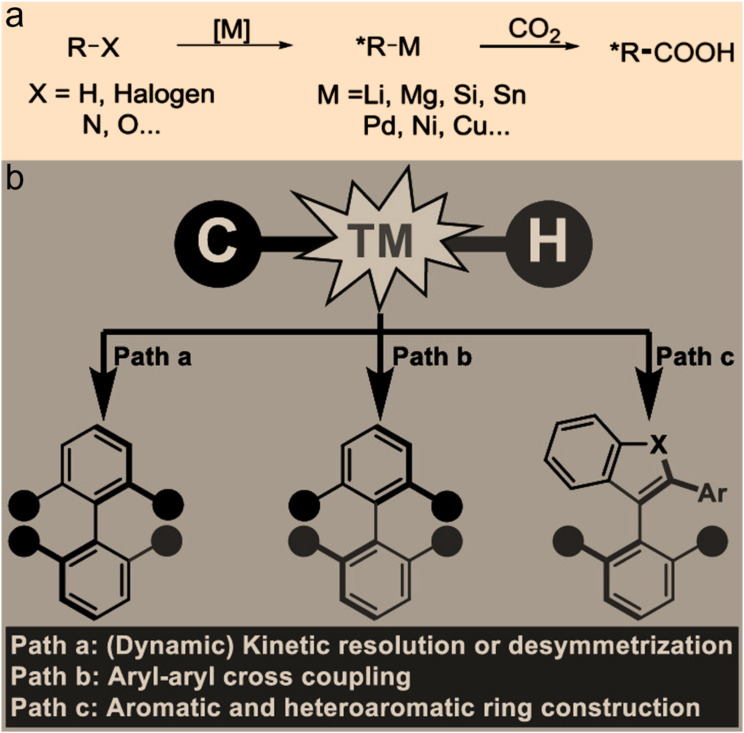
(a) Asymmetric C–C bond formation with CO_2_ (ref. 4). (b) Strategies for enantioselective C–H functionalization (ref. 25).

In a similar way, the formation of asymmetric C–C bonds and asymmetric C–H bonds signifies that the carbon atoms of CO_2_ become chiral after reaction products are formed. Asymmetric C–C and C–H bonds^25^ can be formed directly *via* an enantioselective nucleophilic attack on CO_2_ by organometallic reagents. For example, directly catalyzed asymmetric binding of CO_2_ to unsaturated hydrocarbons is considered as an attractive CO_2_ chemical fixation scheme. Studies have shown that copper-based catalysts can catalyze both the reduction of two prochiral conjugated olefins and the restoration of the product. This allows for enantioselective synthesis of organoboron derivatives with multiple adjacent stereocenters in the presence of diboron compounds.^26^ Another study demonstrated that palladium-based catalysts can catalyze the enantioselective C–H alkylation of allyl groups to yield chiral γ,δ-unsaturated amides.^27^ While progress has been made in the formation of asymmetric C–C and asymmetric C–H bonds, a general method has not been fully developed. It is also worth noting that some nucleophiles possess both N-nucleophilic and O-nucleophilic addition functions. In this case, the formation of asymmetric C–O bonds, asymmetric C–N bonds, and asymmetric C–H bonds may be accompanied by the formation of asymmetric C–C bonds due to the presence of different nucleophilic adducts. The generation of these chiral compounds makes it possible to directly catalyze CO_2_ into chiral food components (Fig. 5).

## The potential of directly converting CO_2_ to chiral food compounds

**Fig. 6:**
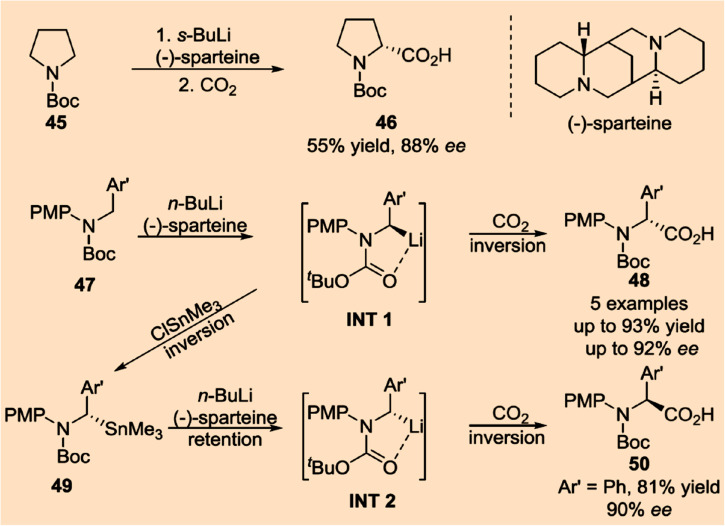
Strategies for direct catalytic conversion of CO_2_ into chiral α-amino acids (ref. 4).

Excitingly, some reports on the synthesis of chiral amino acids from CO_2_ are already available.^21,28^ These pioneering studies confirm that the formation of asymmetric C–N bonds can facilitate the synthesis of α-amino acids with specific configurations. Significant progress has also been made in the asymmetric carboxylation of amines and their derivatives with CO_2_ by using chiral catalysts to synthesize α-amino acids with high *ee* values. On the basis of the asymmetric catalytic function of vitamin B6, Cai *et al.* developed a variety of chiral biomimetic catalytic systems, leading to various chiral amine compounds such as α-amino acids and peptides *via* biomimetic asymmetric transamination and carbonyl-catalyzed α-C–H asymmetric functionalization of primary amines.^21^ Moreover, the obtained α-amino acids can be further polymerized to form high-order chiral poly-amino acids by meticulously selecting specific chiral catalysts (Fig. 6).^28^

Although asymmetric catalytic conversion of CO_2_ has made tremendous progress in the past few decades, direct catalytic conversion of CO_2_ into chiral food components is still challenging. Currently, only a limited number of studies on the synthesis of amino acids and short peptides using CO_2_ exist. The scientific progress in this field is far behind to meet the needs for sufficient food, especially carbohydrates, for the world's growing population. Direct catalytic conversion of CO_2_ to synthesize carbohydrates, functional peptides, and proteins will help relieve the food crisis, but still faces huge obstacles. For example, d-glucose and its aggregates (starch) are the most important carbohydrates utilized by human beings; however, artificial synthesis of d-glucose suffers from complex and tedious separation and purification of chiral configurations. In addition, compared to biocatalysis, the chiral catalysis of carbon dioxide conversion can effectively separate the two enantiomer products by designing the chiral configuration of the catalyst. Thus, the rapid development of asymmetric catalysis based on chiral nanomaterials provides enormous opportunities for the direct conversion of CO_2_ into chiral food components.

## Conflicts of interest

The authors declare no competing interests.

## Supplementary Material
